# Clinical Techniques and Technology: Vestibular Telemetry

**DOI:** 10.1177/0194599821993411

**Published:** 2021-03-02

**Authors:** John S. Phillips, Jacob L. Newman, Stephen J. Cox

**Affiliations:** 1 Norfolk & Norwich University Hospitals NHS Foundation Trust, Norwich, UK; 2 University of East Anglia, Norwich, UK

**Keywords:** Ménière’s disease, migraine, benign paroxysmal positional vertigo, nystagmus, dizziness, vestibular diseases

## Abstract

When a patient presents to a clinician with dizziness, it can be difficult for the patient to describe their symptoms in a clear manner, and clinical examination often yields entirely normal results. Ideally, it would be favorable to measure key physiological parameters during their episodes of dizziness. From a clinical perspective, this would allow a more timely and more accurate diagnosis. From a research perspective, it would allow a greater understanding of how the vestibular system malfunctions as a consequence of vestibular disease. The authors of this report have been funded by the UK Medical Research Council to develop and test a novel technology to measure, record, and analyze key physiological parameters provided by the dizzy individual during an episode of dizziness while active in the community. We provide the context to evolving work in this field, the outcome of preliminary studies, and a consideration of future opportunities.

Dizziness is a common complaint that places a significant burden on health services worldwide.^1^ Dizziness affects 20% to 50% of individuals during their lives, and up to 10% of affected individuals experience vertigo.^2^ In 80% of affected individuals, vertigo results in a medical consultation, interruption of daily activities, or sick leave.^3^

## Vestibular Telemetry

Contemporary methods to evaluate the dizzy patient only provide a snapshot of vestibular function when performed in the absence of a “dizzy attack.” Nystagmus is a key clinical sign that should be documented when assessing patients with vertigo, and various patterns of nystagmus are produced as a consequence of different disease processes. If it were possible to continuously monitor dizzy patients in the community, the presence of a nystagmus pattern could aid diagnosis. We term this diagnostic process *vestibular telemetry*. This approach is analogous to the 24-hour electrocardiogram (ECG) tape used to identify cardiac arrhythmias.

## The CAVA System

**Figure 1.**
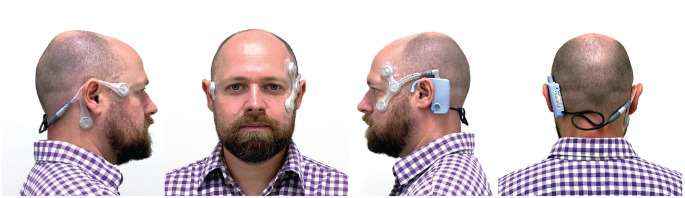
The CAVA (Continuous Ambulatory Vestibular Assessment) device. Two electrodes either side of the eyes record horizontal eye movement, two above and below one eye record vertical eye movement, and a fifth beneath the right ear provides a reference voltage.

The CAVA (Continuous Ambulatory Vestibular Assessment) system consists of a wearable device and computer algorithms to identify nystagmus. The device includes a single-use sensor array that adheres to the face to capture horizontal and vertical eye movements, as well as a reusable module containing an accelerometer, microcomputer, data storage, battery, and connection port (**Figure 1**). Eye movements are recorded via the corneo-retinal potential generated by the eyes. A sampling rate of 42 Hz is used, close to a typical lower end for videonystagmography. This rate reconciles data storage requirements against the level of resolvable detail. Device calibration is not required to identify nystagmus, but an average calibration value is assumed when calculating slow phase velocities.

The CAVA device was developed to allow continuous recording of eye and head movements for 23 hours a day, for 30 days, in the community. Patients remove the device each morning for an hour and then reapply it to themselves. The following findings were obtained from 2 clinical investigations that were reviewed and approved by the National Health Service (NHS) Health Research Authority’s London-Dulwich Research Ethics Committee (IRAS: 261099 and 240847).

We initially tested the CAVA system on a group of healthy individuals to evaluate the suitability of the device and our algorithms’ accuracy at detecting nystagmus induced using an optokinetic video stimulus viewed on a mobile phone.^4^ The algorithms detect the presence of nystagmus using a novel combination of machine learning techniques, and then bespoke analysis routines quantify its more detailed characteristics.^5^ The CAVA system consistently, precisely, and reliably identified periods of induced nystagmus in both stationary and moving subjects with a sensitivity and specificity of 99.1% (95% CI, 95.13%-99.98%) and 98.6% (95% CI, 96.54%-99.63%), respectively. The system could also identify the frequency and beat direction of nystagmus.^5^

Other technologies have been developed to monitor dizziness in the community but have suffered from fundamental limitations, prohibiting continuous wear due to limited data storage, insufficient portability, and inadequate battery life. The CAVA device is small enough to be worn for 30 days, stores more than a month’s worth of data, and requires a single battery change after 15 days. Technologies employing videonystagmography require the eyes to remain open, but patients often close their eyes during vertigo and while asleep. Devices requiring donning or activation upon the onset of dizziness rely on having the device to hand and being physically capable of doing so; challenging for elderly individuals with poor dexterity or those incapacitated by severe vertigo.

## Clinical Applications

**Figure 2.**
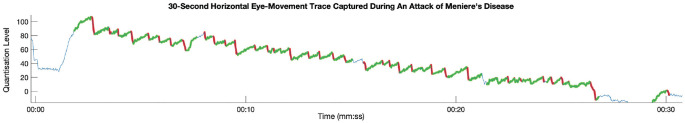
Left-beating nystagmus during an attack of Ménière’s disease. Fast/slow phases are shown in red/green. The attack occurred over about 3 hours and consisted of 8 separate episodes of nystagmus.

**Figure 3.**
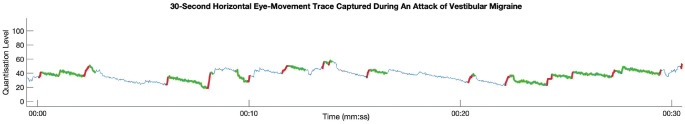
Right-beating nystagmus during a vestibular migraine attack. Fast/slow phases are shown in red/green. Compared to Figure 2, slow phase durations are longer and slow phase velocities are lower. The attack lasted about an hour.

**Figure 4.**
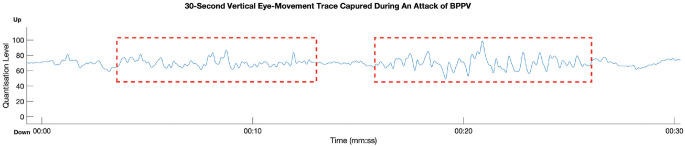
Nystagmus during a benign paroxysmal positional vertigo attack. This nystagmus is oscillatory without obvious fast or slow phases (red boxes). After starting, the nystagmus briefly subsided before resuming again. The duration of the nystagmus was approximately 20 seconds.

We are currently investigating the applicability of the CAVA system in patients reporting vertigo. We have identified quantifiable differences between the nystagmus produced by our target conditions: Ménière’s disease, vestibular migraine, and benign paroxysmal positional vertigo (BPPV).^6^ **Figures 2** to **4** show nystagmus traces produced by these conditions. We have discovered that nystagmus produced during an attack of Ménière’s disease occurs in short episodes lasting several hours, during which the “beat” direction alternates in relation to the affected ear.^7^ By contrast, nystagmus during a vestibular migraine attack is shorter in duration, and its slow phase velocities are generally lower. The nystagmus of BPPV is even shorter in duration and is induced by acceleration of the head, as confirmed by CAVA’s accelerometer signals.

We have recorded an entire episode of vertigo in a patient with Ménière’s disease.^7^ Because we recorded eye movements before, during, and after the attack, we have discovered a possible prodromal phase. If this is a consistent feature of Ménière’s disease, it could be exploited to warn patients of an impending attack. In another study, we analyzed nystagmus traces from different patients with Ménière’s disease, revealing how the characteristics of nystagmus could aid decision making with respect to treatment.^8^

## Future Developments

The CAVA system fulfills an unmet clinical need to provide a long-term, objective record of a patient’s vertigo. Such a record could also be used to confirm reports of “dizziness” following work-related head injuries or road accidents. The CAVA device has the potential to aid the diagnosis and understanding of many areas of vestibular medicine, conditions outside the vestibular system, nonvestibular areas of medical research (eg, sleep medicine), and beyond (eg, driver alertness monitoring).

The diagnosis of Ménière’s disease and vestibular migraine is contentious. Many tests are often required, including radiological investigations, audiometry, and other specialist vestibular tests. As more nystagmus data become available, in addition to identifying nystagmus patterns that are consistent with conditions such as Ménière’s disease and vestibular migraine, it might be possible to create universal diagnostic criteria for these conditions, as well for disease subtyping, grading, and staging.
